# 重视并规范我国血液系统疾病实验室自建检测

**DOI:** 10.3760/cma.j.issn.0253-2727.2023.01.001

**Published:** 2023-01

**Authors:** 慧君 王, 琦 孙, 志坚 肖

**Affiliations:** 1 中国医学科学院血液病医院(中国医学科学院血液学研究所)，实验血液学国家重点实验室，国家血液系统疾病临床医学研究中心，细胞生态海河实验室，天津 300020 National Clinical Research Center for Blood Diseases, State Key Laboratory of Experimental Hematology, Haihe Laboratory of Cell Ecosystem, Institute of Hematology & Blood Diseases Hospital, Chinese Academy of Medical Sciences & Peking Union Medical College, Tianjin 300020, China; 2 天津医学健康研究院，天津 301600 Tianjin Institutes of Health Science, Tianjin 301600, China

目前服务于临床及患者的体外诊断产品主要有两类：一类为经过药品监管部门审批注册的体外诊断（In Vitro Diagnostic, IVD）产品，包括医疗器械和诊断试剂；另一类是由实验室自行研发、验证且只在实验室内部使用的实验室自建检测（Laboratory Developed Tests, LDT），可以作为IVD的补充。即使是对国家药品监督管理局（National Medical Products Administration，NMPA）批准的IVD产品进行修改，改变制造商关于预期用途的临床声明，亦将构成一个新的LDT。LDT不作为商品出售给其他实验室、医院及个人[1]–[2]。我院病理中心已开展的血液系统疾病诊断检测项目共361项，其中IVD 96项，其他属LDT。

血液系统疾病诊断相较其他系统疾病对实验诊断的依赖性更高，近年，一些基于细胞遗传学和分子学等发病机制的疾病亚型得以确认，加入分子学的疾病预后积分系统不断提出，针对致病基因的小分子靶向药物和疾病起始克隆异常表达抗原单（双）克隆药物的临床试验的开展和完成上市，开启了个体化医疗的新时代，LDT具有技术更新快、从实验室向临床转化快等特点，因而，在精准诊治新时代愈显重要。

LDT领域我国相较欧美及日本等亚洲国家起步较晚，直到2017年才由中华医学会检验医学分会发表了“我国医学检验部门自建检验方法发展与管理建议”[3]，2021年2月9日发布的国务院793号令，即“医疗器械监督管理条例”中的第五十三条规定：对国内尚无同品种产品上市的体外诊断试剂，符合条件的医疗机构根据本单位的临床需要，可以自行研制，在执业医师指导下在本单位内使用。对LDT项目监管不到位，将会对患者的权益造成潜在的风险，但过度监管又会阻碍临床精准诊疗工作。因此管理部门如何平衡其中的权益，医疗及检测机构如何做好实验室质量管理、规范操作流程，对于提高血液系统疾病的诊治水平、促进学科发展至关重要。

一、LDT在血液系统疾病诊断中的重要性

血液系统疾病是与实验诊断关系最密切的一类疾病：其一，血液系统疾病的诊断、分类、预后分层体系不断进展；其二，随着新型化学及靶向药物的开发和应用，在靶点筛选、疗效评估及预后判断方面都对临床检测提出更高要求。从2022版WHO关于淋巴及造血组织疾病的分类更新和髓系肿瘤、急性白血病国际共识分型中可以看出，越来越多的疾病亚型都是基于基因学结果，全基因组测序、外显子测序越来越多地应用于临床[4]–[5]。此外，有些先天（遗传）性血液病诊断项目临床应用数量较少，试验对象较为局限，难以达到临床性能验证要求而无法按照传统的上市监管得到批准，这些都是LDT在血液病诊疗中广泛应用的基础。

血液病诊断离不开最基本的形态学、免疫学、细胞遗传及分子遗传学诊断试验，其中流式细胞技术、荧光原位杂交（FISH）及第二代测序技术（Next Generation Sequencing, NGS）等众多检测项目都以LDT模式为主。如NGS，该产品检测步骤繁多，技术复杂，涉及生物信息分析、报告解读等多方面人才；同时对实验室布局、生物安全、设备运行成本都有较高要求。涉及流式细胞技术的检测项目缺乏严格意义的质控物和标准品，结果分析更多地依赖操作者的经验，这些都要求技术人员具有较高的专业能力，结果的解释应具有临床权威性。

美国食品药品监督管理局（FDA）将LDT分为三类：FDA注册/批准后实验室进行了修改的试剂或检测系统；未经FDA注册/批准的试剂或检测系统；未提供性能指标的试剂或检测系统[6]。血液病分子诊断中涉及的PCR和高通量测序等绝大多数产品，如引物、探针、扩增缓冲液、酶等均属于此类。

二、国外监管经验

纵观全球范围内，在LDT的监管和实施方面最多的经验来源于美国。据美国疾病控制和预防中心（CDC）估计，全美LDT的总量在60 000～100 000项，有15 000～20 000家实验室有能力开发复杂的LDT。美国对LDT的监管采用政府机构和认证机构协同管理模式。由联邦政府部的医疗保险和辅助服务中心（CMS）和FDA，分别依据临床实验室改进修正案（The Clinical Laboratory Improvement Amendments, CLIA）和医疗器械修正案（MDA）共同监管，并不需要FDA的批准。CMS主要对实验室进行CLIA法规监管，确保实验室有能力进行精准、可靠的检测。只有经过CLIA认证的实验室才可以提供人体样本的检测，具备CLIA资质的临床实验室，就可以开展LDT。FDA主要对实验室的具体检测方法进行监管，对测试项目的准确性和精度进行更加细致的评估，在上市前对检测项目的临床有效性进行确认。CMS授权的认证机构有实验室认证委员会（COLA）、美国病理学家协会（CAP）和联合委员会（TJC）等共七家。由CMS审核通过的实验室获得“合规证书”, 而由认证机构如CAP审核通过的实验室获得“认可证书”。

随着检测技术与商业模式的发展，LDT已经从传统模式进展到现代LDT模式，包括已广泛用于筛查常见疾病、干预治疗决策，技术流程高度复杂等，这些变化使得LDT的潜在风险大幅提高。同时，CMS根据CLIA'88的监管侧重于评估实验室检测能力，并未对上市前后LDT的临床有效性、安全性等进行评估。因此，FDA开始考虑对LDT进行监管，并于2014年10月发布了LDT监管框架指南（草案），将LDT作为IVD的一个子集进行监管[7]。但该草案遭到了众多学术团体、行业协会及生物科技公司的反对，认为FDA不具备监管LDT的法定资格，同时还会阻碍新项目的开展和创新。2016年FDA暂停了实施指南草案的计划。国会于2017年发布了《诊断准确性和创新法案》（DAIA），2018年底发布了《验证准确的前沿IVCT开发法案》（VALID），旨在将IVD和LDT统称为体外临床检测（IVCT），并由FDA监管[8]–[9]。此外，在2020和2021年提出的《美国实验室验证创新检测法案》（VITAL）主张将LDT脱离FDA的监管，得到美国临床化学协会（AACC）的支持。

欧盟与日本均无明确的LDT监管定义及规范。欧盟各国对LDT监管要求不同，不受体外诊断医疗器械指令监管，日本也无LDT相关管理政策，许多医学检验部门研发的设备和试剂未经审批已在临床应用。

三、国内现状

早在2013年9月，原国家卫生计生委医政管理局就批准成立“国家卫生计生委个体化医学检测试点单位”（也称为“LDT试点单位”）。NMPA 2021年3月发布并从6月1日起实施《医疗器械监督管理条例》，其中第五十三条规定：对国内尚无同品种产品上市的体外诊断试剂，符合条件的医疗机构根据本单位的临床需要，可自行研制，在执业医师指导下在本单位内使用。新《条例》明确了LDT的定义、范围界定及管理模式，规定LDT的建立和应用要符合三个条件：①国内尚无同品种产品上市的体外诊断试剂；②符合条件的医疗机构；③自行研制项目仅限于在本单位内使用。2021年7月，国务院发布的《关于支持浦东新区高水平改革开放打造社会主义现代化建设引领区的意见》中提出：在浦东新区范围内允许有条件的医疗机构, 按照相关要求开展自行研制体外诊断试剂试点，NMPA相关分局已经启动有关的LDT的监管政策研究。

四、LDT实验室的准入管理建议

对LDT的监管建议采用卫生健康委员会与药品监督管理局共同监管的管理模式，可参照美国的CLIA认证模式，对开展LDT的实验室实行准入管理，获得资质的实验室可自主进行LDT。考虑到国内实验室现状，起步阶段可以利用现存的国际通行评价体系进行准入管理，如通过CLIA、CAP或中国合格评定国家认可委员会（CNAS）认可的三甲医院检验部门或第三方独立检验实验室可优先开展LDT[10]。其中CLIA和CAP要求全员、全项目覆盖，是对实验室整体能力的认可，而CNAS目前已不再受理LDT项目，因此建议在准入方面优选具备CLIA或CAP认可证书的医疗机构或第三方实验室。我国监管能力提升后可逐步过渡至国内第三方认证或监管部门直接认证。

LDT实验室申报形式可有以下三种模式：①具备LDT产品研发能力的公立医院；②以第三方医学检验实验室为主体，与暂不具备独立研发孵化能力的三级医院联合进行LDT实验室资质申报；③具备独立研发孵化LDT产品能力且具备对LDT产品检测结果做出专业解释的第三方医学检验实验室。

在CLIA框架下，FDA根据LDT项目的复杂性将其分为三类：豁免试验、中度复杂试验、高度复杂试验，后两者并称为非豁免试验。国内可采用注册及备案制，低风险LDT实行产品备案管理，中、高风险LDT实行产品注册管理。注册流程包括性能评价、实验室认证、生产环节质量管理体系认证三个部分。备案流程是由备案人向市级药品监督管理部门提交备案资料，备案待查。

五、质量管理体系要求

通过LDT认证的实验室意味着从研发、检测到结果解释的全过程体系都经过充分、严格的性能确认和评估，具备开展自建检测项目的能力。

参考ISO15189《医学实验室质量和能力认可准则的应用要求》，医学实验室应按照质量管理体系运行并具有技术能力。其分为管理要素15条：组织与管理、质量管理体系、文件控制、服务协议、委托实验室的检验、外部服务和供应、咨询服务、投诉的解决、不符合项的识别和控制、纠正措施、预防措施、持续改进、记录控制、评估和内部审核。技术要素10条：人员、设施和环境条件、设备/试剂和耗材、检验前程序、检验程序、检验结果质量保证、检验后程序、结果报告、结果发布和实验室信息系统。

CAP认证是美国病理学家协会组织的针对医学实验室开展的一种国际项目认证，也是对实验室技术管理水平的全面认可，通过该认可意味着诊断质量与水准进入国际最高水平行列，并获得国际相关机构认同。CLIA认证由美国临床实验室委员会颁发实验室资质证书，表明该实验室符合美国联邦政府实验室修正案的相关规定，达到优质实验室标准，CLIA认证也代表了目前国际最高水平的认证标准。以上认证同样要求实验室具备完善的质量管理体系，并渗入到日常管理过程中。

1. 人员资质：人员是实验室最重要的组成部分，进行非豁免实验的实验室岗位包括实验室主任、技术主管、检测人员和临床顾问等，需要根据相关岗位要求及检测项目的专业性获取相关资格证书。确保有足够的经过适当培训的人员是实验室主任最重要的职能之一。CLIA要求在检测患者标本之前，所有人员必须接受适当的培训和考核，尤其要重视新员工的能力评估[11]。

2. 实验室资质：实验室的软硬件资质需要满足实验室所开展项目对应的条件要求。同时实验室的安全性须符合要求，具体体现在生物安全、化学安全、放射安全和医疗废物管控等；实验室信息系统（Laboratory Information System, LIS）所有设备均需要维护，在使用前需对新的硬件和软件进行确认，能够生成最终检测报告和初始报告的备份，在无授权的情况下不可随意使用LIS数据。

3. 临床评估：LDT在应用于临床诊疗前应对预期用途进行沟通，包括疾病风险分层、疾病筛查、疾病鉴别或辅助诊断、预后及疾病状态监测等，临床预期用途可以预先设定，但最终根据性能确认结果得出结论。同时还要了解临床对实验室的要求，包括检测频次、报告发放时间等，以及检测局限性。

4. 项目性能确认：LDT投入临床检测前需完成方法学性能确认，这是LDT中最关键的部分，确保新建方法能满足性能要求，并且得到准确、可靠、可重复的结果。CAP和ISO15189不同认可体系对方法学确认都有规定，根据国际临床和实验室标准化协会（CLSI）文件，需要分析确认的性能参数包括正确度、精密度、线性范围、参考区间、分析灵敏度，以及试剂和样品的稳定性等。如实验室接受多种类型的标本，对每种标本类型均需分别进行评价。不同专业、不同数据分析类型的检测项目确认的参数可不同。

由于NGS检测位点较多，无法对所有检测位点都进行确认，可以选取不同的突变类型（单核苷酸变异、缺失、拷贝数变异和结构变异）和临床意义明确的常见基因进行确认。某些罕见病或遗传性疾病，如凝血因子缺乏、家族性噬血细胞综合征等由于在方法确认初期，阳性标本数量受到限制，对这类疾病的确认无法按照规定要求的例数完成，可以考虑适当放宽标准，随着后期数据的不断积累，对其确认参数进行持续性评估，对方法进行不断优化和改进。

流式细胞术方法学性能确认的难度较大，原因有细胞检测的复杂性、多种疾病状态下样本背景差异、缺乏参照物、缺乏校准曲线，使流式细胞术不能复制生化等可溶性检测物的准确性及线性验证方法。2021年10月由CLSI制定了《流式细胞术方法学确认指南-第1版》（CLSI-H62）,旨在给出共识性的流式细胞术检测全流程质量验证指导建议[12]。指南提出了一种适用分析（fit-for-purpose，FFP）方法确认，即根据数据的预期用途及相关监管要求进行调整的分析确认方法。其中选择性、精密度、检测能力、参考区间和稳定性相对容易确认，准确性和线性确认具有挑战性。首先根据项目类别及生物分析数据类型确定参数，然后制定确认计划，随后进行确认实施、制定确认报告。以白血病/淋巴瘤免疫分型为例，检测项目为定性实验，疾病的存在与否由异常/肿瘤细胞群的存在与否来确定。其准确度的评估可以依赖诊断一致性方法，该方法更接近“临床准确度”，而不是“分析准确度”，也可采用参考/对比方法一致性进行确认。定性检测不需要对线性进行确认。目前流式细胞术检测均为多参数，很多较大实验室在样本制备中都采用抗体预混的方法，预混试剂的稳定性同样需要确认。

5. 标准化操作流程：方法学建立并确认后，应制定详细的标准化操作流程（SOP），以保证临床检测的正常进行，对所有试剂性能、标本类型、运输和储存、样本处理、检测、结果判断、临床意义等内容均需详细规定。如有可影响检测结果的分析软件均需在SOP中注明。SOP一旦制定，应严格遵照执行并定期更新，如发现SOP存在缺陷，实验室应经过讨论、验证后及时修改。

6. 室内质控及室间质评：室内质控是检测结果重复性和准确性的重要保证。定性检测项目室内质控要求为每批次实验应设置阴性、弱阳性和（或）阳性质控物，定量检测项目室内质控要求为每批次实验设置高、中、低质控物，分子检测项目，还应设置阴性质控，监控是否存在污染[13]。由于绝大多数LDT项目都没有商品化的室内质控品，如为自制室内质控品，必要时需定期评估其稳定性。白血病/淋巴瘤免疫分型中，对于某些正常细胞不表达，仅表达于少数类型的肿瘤细胞抗原，如CD1a、CD103等，检测抗体的有效性不易评估，CAP的能力验证中有相应的项目可以参加。

所有LDT实验室均需参加室间质评，以验证其检测能力。使用可代表预期临床检测样本的样本类型进行验证，评估分析性能。实验室进行质评时需要使用与临床实际检测时一致的仪器，纳入实验室常规工作流程。样本不可重复检测，在实验室向能力验证机构报告结果规定日期之前，不得参与与能力验证样本结果相关的实验室间交流或讨论。结果回报后应对结果进行总结，能力验证计划中获得的分数小于100％的结果应分析根本原因。

对于成绩不合格的医疗机构所开展的LDT需要停止检测，整改后需对再次提交的LDT性能验证数据进行审查。审查合格后，方可继续开展。

六、结语

LDT在血液病诊疗中占有重要地位，借鉴国外监管政策和经验，我国也于近年来出台了相关政策，希望在此背景下，有相关认证资质的大型医疗机构及第三方实验室能够在完善的质量管理体系下，不断研制开发出顺应学科发展并适合临床需求的、能够提高血液病诊疗水平的LDT项目。但现今在诸如LDT方法学性能和临床性能确认标准、开展LDT实验室的质量管理体系标准、LDT使用试剂和检测系统标准、如何管理和具体由哪些部门管理等尚待制定和（或）细化明确，行业协会可以在LDT的标准化和规范化等方面发挥学术引领作用，在精准医疗的新时代，LDT定将造福于社会和血液病患者。
